# Impact of an evidence‐based sepsis pathway on paediatric hospital clinical practice: A quality improvement study

**DOI:** 10.1111/1742-6723.70036

**Published:** 2025-04-07

**Authors:** Bernard McCarthy, Natalie Middleton, Fenella J Gill, Zoy Goff, Zoe Paterson, Christopher C Blyth, the CAHS Sepsis Working Group, Kathleen Anastasas, Gabrielle Anstey, Michael Baker, Sarah Cherian, Michael Collin, Catherine Dunstan, Simon Erickson, Katherine Griffiths, Pania Falconer, Tim Ford, Kellie Francis, Dimple Goel, Craig Hasler, Ashleigh Kenworthy, Michal Levitt, Ariel Mace, Vaanitha Manickavasagar, Andrew Martin, Lauren O'Conner, Joel Parke, Marianne Phillips, Melanie Robinson, Andrew Savery, Scott Stokes, Jessica Spragg, Kate Wheadon, Eliza Wziontek

**Affiliations:** ^1^ Department of Paediatric Emergency Medicine Perth Children's Hospital, Child and Adolescent Health Service Perth Western Australia Australia; ^2^ Child and Adolescent Health Service Sepsis Program Perth Children's Hospital, Child and Adolescent Health Service Perth Western Australia Australia; ^3^ School of Nursing Faculty of Health Sciences, Curtin University Perth Western Australia Australia; ^4^ Department of Infectious Diseases Perth Children's Hospital, Child and Adolescent Health Service Perth Western Australia Australia; ^5^ Division of Paediatrics School of Medicine, Faculty of Health and Medical Sciences, The University of Western Australia Perth Western Australia Australia; ^6^ Wesfarmers Centre of Vaccine and Infectious Diseases The Kids Research Institute Australia, Perth Children's Hospital Perth Western Australia Australia; ^7^ Department of Microbiology PathWest Laboratory Medicine, QEII Medical Centre Perth Western Australia Australia

**Keywords:** audit, paediatric, quality improvement study, sepsis clinical care standard, sepsis pathway

## Abstract

**Objectives:**

To assess the impact of implementing a sepsis pathway and education program on key sepsis outcomes and performance targets in a tertiary paediatric hospital.

**Methods:**

A quality improvement study using a multi‐modal screening process and pragmatic clinical definitions. Treatment of all children with septic shock and sepsis without shock 4 months prior to pathway/education package launch was compared with those meeting definitions 8 months post‐launch.

**Results:**

Over the study period, 1483 episodes were screened; 517 episodes met study definitions (171 pre‐launch; 346 post‐launch). Eighty‐two episodes met septic shock definitions (15.9%) and 435 met sepsis without shock definitions (84.1%). A total of 143 episodes pre‐launch and 271 episodes post‐launch were managed exclusively at Perth Children's Hospital (PCH). Post intervention, the pathway form was utilised in 146 of 271 episodes (53.9%). Pathway/education package introduction was associated with a reduction in the median time from recognition to antibiotic administration (60 [IQR: 26; 115] to 45 min [IQR: 16; 75] for those with septic shock and/or sepsis without shock treated exclusively at PCH; *P* < 0.001). The proportion receiving antibiotic therapy within recommended timeframes significantly increased (septic shock within 60 min: 70.0% to 92.5%, *P* < 0.03; sepsis without shock within 180 min; 86.2% to 94.8%, *P* = 0.005). No statistically significant change in length of stay, intensive care admission, mortality or antibiotic consumption was observed following pathway launch.

**Conclusions:**

Paediatric sepsis pathway and education package implementation can reduce time to antibiotics in sepsis and aid local data collection and surveillance of patients treated for sepsis.

## Graphical abstract

A quality improvement study at a Western Australian tertiary paediatric hospital found that implementing a paediatric sepsis pathway and associated education program can reduce the time to administer antibiotics in sepsis cases. A pragmatic audit supports local data collection and enables effective surveillance of sepsis patients.

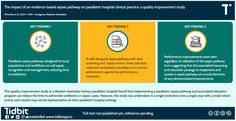

**Key findings**

- Implementation of a paediatric sepsis pathway, designed for local populations and workflows and incorporating screening, recognition, and management tools, can lead to a reduction in time to antibiotics in sepsis.
- A well‐designed pathway with clear screening and ‘sepsis review’ time aids data collection and patient surveillance to monitor performance against key performance indicators.
- Performance improvements were seen regardless of the utilisation of the paper pathway form, suggesting that the associated resourcing and education package to implement and sustain a pathway are crucial elements of any demonstrated improvements.

## Introduction

Sepsis is a dysregulated host response to infection leading to life‐threatening organ dysfunction. Septic shock is sepsis with evidence of cardiovascular dysfunction.^1^, ^2^ It is associated with high morbidity and mortality and is the leading preventable cause of childhood death and disability.^3^, ^4^, ^5^

The Surviving Sepsis Campaign recommends early treatment with a bundle of interventions to improve outcomes.^6^ Key interventions are the administration of appropriate antibiotic therapy and the reversal of shock. Weiss *et al*. found that mortality increased if antimicrobials were delayed beyond 3 h in the paediatric population.^6^

A major barrier to early intervention is that sepsis can be challenging to recognise and is rapidly progressive.^7^ International guidelines recommend the development and implementation of local sepsis pathways and recognition tools.^6^ In June 2022, the Australian Commission of Safety and Quality in Health Care (ACSQHC) Sepsis Clinical Care Standard^8^ was released recommending that all healthcare services have a sepsis governance process, a sepsis coordinator and a locally approved sepsis pathway, with an audit process to monitor key performance indicators (KPIs). A competency‐based training program on the use of the pathway was also recommended. Modelling in a comparable Australian healthcare setting has suggested that pathways can moderately reduce healthcare costs.^9^ At Perth Children's Hospital (PCH), a sepsis program was established in July 2022 with the appointment of a medical lead and clinical nurse consultant. Initial priorities were the implementation of a site‐specific sepsis pathway, education program and audit tool designed to capture patients treated for sepsis and monitor key outcome measures and performance targets (hereafter referred to as the *pathway program*).

We describe the development of the pathway program and assess the impact following its launch, testing the hypothesis that the implementation of a site‐specific sepsis pathway and education program would improve KPIs and key timed sepsis outcomes.

## Methods

This quality improvement study uses data collected from patients treated for sepsis with or without shock at PCH in the 4 months prior to pathway program launch (14 October 2022 to 19 February 2023) and data from the 8 months post‐launch (20 February to 31 October 2023). The medical records reviewed for this period were paper‐based, encompassing both medication charts and clinical notes. A STROBE Statement checklist of items for observational studies was followed (Supplement 1 in the Supporting Information).

### Population

Located in Western Australia (WA; population 2.9 million), PCH is the state's only tertiary paediatric hospital and trauma centre. PCH provides care to children from birth until 16 years of age, with 298 inpatient beds, approximately 30 000 admissions and 70 000 emergency attendances per year.^10^ All children aged 0–16 years treated for sepsis with or without shock (Box 1) at PCH were eligible for inclusion; neonates managed exclusively within the neonatal intensive care unit or transferred from maternity hospitals directly to the neonatal unit were excluded.

### Pathway program

The PCH Sepsis Pathway (Supplement 2 in the Supporting Information) was developed by the Child and Adolescent Health Service (CAHS) Sepsis Working Group to improve sepsis recognition, escalation, and multidisciplinary care and aid discharge planning. It was designed to be used hospital‐wide, with the exclusion of the neonatal intensive care unit. The pathway was developed as a screening/recognition, escalation, and audit tool incorporating documentation of a targeted clinical assessment (a ‘sepsis review’) and a priority‐based resuscitation guide capturing key time‐based interventions. The pathway was developed to align with the pre‐existing PCH Sepsis Recognition and Management Guideline and the statewide paediatric ESCALATION System that includes observation and response charts with an embedded sepsis recognition tool.^11^, ^12^

In accordance with the ACSQHC Sepsis Clinical Care Standard^8^ the education package is a competency‐based training tool. Prior to the sepsis pathway launch, >80% of PCH doctors and nurses completed the education program, either at an in‐person workshop or *via* the e‐learning module. The education program was incorporated into all hospital induction programs for nurses and doctors and is part of PCH essential training requirements.

### Sepsis audit tool

As there was no sepsis registry at PCH and sepsis was inconsistently documented and coded within medical records and administrative databases,^13^ a comprehensive local screening system was developed. This screening system was designed to be inclusive, enabling identification of all children with suspected sepsis/septic shock.

Children with ‘potential sepsis’ were identified *via* the following methods:

- Hospital discharge diagnostic codes (ICD‐10‐AM 12th Ed.) sepsis codes^14^ (Supplement 3 in the Supporting Information)
- Australian and New Zealand Paediatric Intensive Care Registry^15^ episodes of coded sepsis or septic shock.
- Safety Team Afterhours Response Service Clinical Nurse Specialist daily handover lists (searching for key words including ‘suspected sepsis’, ‘sepsis’, ‘septic shock’, ‘severe infection’, ‘antibiotics’, ‘fluid bolus’, ‘patient of concern’).
- Daily review of admissions through hospital administrative databases undertaken in collaboration with the Paediatric Active Enhanced Disease Surveillance Program^16^ (searching for key words including ‘sepsis’, ‘septic shock’, ‘query sepsis’, ‘suspected sepsis’, ‘bacteraemia’).
- Daily review of paediatric critical care unit (PCCU) admissions
- Review of in‐hospital deaths

Two additional methods were utilised as part of the pre‐launch cohort but were not continued as they did not yield additional cases:

- CAHS Bacteraemia Database
- Reports of antibiotics dispensed in the PCH ED.

Patient records of children identified through the screening process were reviewed by the sepsis clinical nurse consultant (NM) to determine if eligibility criteria were satisfied. Data extraction was conducted by NM with BMc and CCB assisting when additional clinical clarification was required or when the time of sepsis recognition was unclear.

### Pragmatic sepsis definitions

The present study was undertaken prior to the publication of the Phoenix Criteria^15^ for paediatric sepsis and septic shock. At the time of the study, the existing IPSCC paediatric SIRS‐based sepsis definitions were known to commonly identify children with mild illness and lack specificity for sepsis‐related mortality.^17^, ^18^ The limited number of suitable screening tools available^19^, ^20^ prompted the development of a novel tool with pragmatic clinical definitions (Box 1).

### Audit data collected

Demographic variables, risk factors and outcomes were collected following review of hospital administrative and clinical records. Time and results of the first lactate and blood culture post‐sepsis recognition were collected from laboratory records. Antibiotic dispensing data were obtained from the hospital pharmacy and compared to whole‐of‐hospital and ED activity.

### Quality measures and ‘time point zero’

The KPIs measured (in accordance with ACSQHC Sepsis Clinical Care Standard) included: (i) time to antibiotics from recognition; (ii) lactate taken during screening; (iii) blood cultures taken during management; (iv) documentation of sepsis treatment in the patient's discharge summary; (v) adherence to local paediatric sepsis recognition and management guidelines; and (vi) utilisation of the sepsis pathway form (post‐launch). These KPIs are only reported for the cohort managed initially at PCH and not for patients transferred from other hospitals after commencement of care for sepsis.

Monitoring KPIs in sepsis required identifying a ‘time point zero’. Previous studies have used a range of thresholds^21^ incorporating blood pressure^22^ and heart rate measurement.^23^ Surviving Sepsis Paediatric recommendations for antimicrobial therapy use time of recognition of sepsis/septic shock as ‘time point zero’.^6^ For the study, a pragmatic ‘time zero’ was defined as: the time a clinician first documented an impression of sepsis with or without shock in the clinical notes or pathway or the documented time the clinician performed a sepsis review prior to treatment. If this was not available, the time recorded in nursing notes or triage assessments requesting a sepsis review was used. If neither was documented, it defaulted to the time that criteria were met for a sepsis review based on trigger of the sepsis recognition tool (which includes observation chart, clinical and lactate/blood glucose level triggers).

### Data analysis

Descriptive analysis was conducted summarising demographics, risk factors, performance indicators and outcomes. Categorical variables were reported as frequency and proportions with differences assessed using Chi‐squared analyses. Continuous variables were reported using mean and standard deviations. Normally distributed data were assessed using t‐tests while non‐parametric data were assessed using Mann–Whitney tests. Trend data was assessed using box‐whisker plots divided in two‐month intervals. Data analysis was performed using Excel365 and STATA18.

### Ethical considerations

The study was approved as a quality improvement activity by the Child and Adolescent Health Service Governance, Evidence, Knowledge, Outcomes (GEKO) committee, project 2023‐000448.

## Results

**Figure 1:**
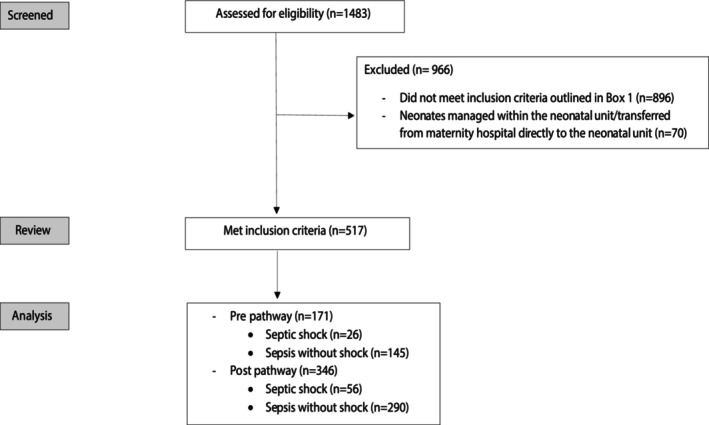
Flow diagram of patient inclusion.

Over the study period, 1483 episodes were screened: 517 met study definitions (hereafter referred to as total cohort), 171 pre‐pathway, 346 post‐pathway (Fig. 1; Table 1). This included 82 (15.9%) meeting septic shock definitions and 435 episodes meeting sepsis without shock definitions. This equated to approximately 10 patients/week managed at PCH for sepsis and approximately two children per week admitted to the PCCU, with a similar incidence pre‐ and post‐pathway program.

**TABLE 1:** Demographics and risk factors for the total cohort, those with septic shock and sepsis without shock, pre‐ and post‐pathway program

	Total cohort (*n* = 517)			Septic shock (*n* = 82)			Sepsis without shock (*n* = 435)		
	Pre‐pathway	Post‐pathway	Significance	Pre‐pathway	Post‐pathway	Significance	Pre‐pathway	Post‐pathway	Significance
	*n* = 171	*n* = 346		*n* = 26	*n* = 56		*n* = 145	*n* = 290	
Demographics									
Age *years*, median (IQR)	0 (0, 3)	1 (0–4)	NS	3 (1, 12)	3 (0, 5.5)	NS	1 (0, 4)	0 (0, 3)	NS
Male, Gender	100 (58.5%)	190 (54.9%)	NS	15 (57.7%)	26 (46.4%)	NS	85 (58.6%)	164 (56.6%)	NS
Aboriginal and/or Torres Strait Islander	20 (11.7%)	47 (13.6%)	NS	7 (26.9%)	6 (10.7%)	NS	13 (9.0%)	41 (14.4%)	NS
Site of escalation of care, PCH	143 (83.6%)	271 (78.9%)	NS	20 (76.9%)	40 (71.4%)	NS	123 (84.8%)	231 (79.7%)	NS
Other HSP and then transfer to PCH	28 (16.4%)	75 (21.7%)	NS	6 (23.1%)	16 (28.6%)	NS	22 (15.2%)	59 (20.3%)	NS
Risk factors									
Infants less than 3 months	42 (24.6%)	108 (31.2%)	NS	1 (3.9%)	10 (17.9%)	NS	41 (28.2%)	98 (33.8%)	NS
Immunosuppression, asplenia, chemotherapy, long‐term steroids	8 (4.7%)	29 (8.4%)	NS	1 (3.8%)	5 (8.9%)	NS	7 (4.8%)	24 (8.3%)	NS
Invasive devices	11 (6.4%)	36 (10.4%)	NS	2 (7.7%)	9 (16.1%)	NS	9 (6.2%)	27 (9.3%)	NS
Recent surgery, burn or wound	6 (3.5%)	22 (6.4%)	NS	0	6 (10.7%)	NS	6 (4.1%)	16 (5.5%)	NS
Unimmunised or incomplete immunisation	7 (4.1%)	56 (16.2%)	*P* < 0.001	2 (7.7%)	8 (14.3%)	NS	5 (3.4%)	48 (16.6%)	*P* < 0.001
Remote, delayed access to healthcare or patient transfer	13 (7.6%)	51 (14.7%)	*P* = 0.02	3 (11.5%)	10 (17.9%)	NS	10 (6.9%)	41 (14.1%)	*P* < 0.03
Representation (including GP)	27 (15.8%)	40 (11.6%)	NS	5 (19.2%)	7 (12.5%)	NS	22 (15.2%)	33 (11.4%)	NS
Family and/or clinician concern	15 (8.8%)	20 (5.8%)	NS	3 (11.5%)	4 (7.1%)	NS	12 (8.3%)	16 (5.5%)	NS
Culturally and or linguistically diverse	20 (11.7%)	59 (17.1%)	NS	5 (19.2%)	10 (17.9%)	NS	15 (10.3%)	49 (16.9%)	NS
Recent sepsis diagnosis	2 (1.2%)	9 (2.6%)	NS	0	0	NS	2 (1.4%)	9 (3.1%)	NS
Complex/chronic medical condition	33 (19.3%)	57 (16.5%)	NS	9 (34.6%)	15 (26.8%)	NS	24 (16.6%)	42 (14.5%)	NS

GP, general practitioner; HSP, health service provider; IQR, interquartile range; PCH, Perth Children's Hospital.

### Demographics

Children with septic shock had a median age of 3 years (IQR: 0, 7.25) whereas children with sepsis without shock had a median age of 1 year (IQR: 0, 3; Table 1). Aboriginal and/or Torres Strait Islander (hereafter Aboriginal) children comprised 15.9% (*n* = 13) of those with septic shock and 12.6% (*n* = 55) of those without shock. Approximately 20% of episodes, in both the pre‐ and post‐pathway groups, were managed at another hospital prior to transfer to PCH. Of those initially managed at PCH (*n* = 414), 84.1% met criteria in the ED, the remainder on the ward.

### Interventions, microbiology and outcomes

The median fluid resuscitation volume during the first hour was 30 mL/kg for septic shock and 10 mL/kg for sepsis without shock (Table 2). Forty‐six children in the total cohort (8.9%) received vasoactive medications and 48 (9.3%) required intubation. Overall, 116 episodes required admission to PCCU (22.4%), including 54 (65.9%) of the septic shock group. No significant differences were observed in any of these interventions following pathway introduction.

**TABLE 2:** Interventions and outcomes in the total cohort, those with septic shock and sepsis without shock, pre‐ and post‐pathway program

	Total cohort (*n* = 517)			Septic shock (*n* = 82)			Sepsis without shock (*n* = 435)		
	Pre‐pathway	Post‐pathway	Significance	Pre‐pathway	Post‐pathway	Significance	Pre‐pathway	Post‐pathway	Significance
	*n* = 171	*n* = 346		*n* = 26	*n* = 56		*n* = 145	*n* = 290	
Interventions – All									
Fluid bolus (mL/kg); median; (IQR)	10 (10, 12.6)	10 (0, 20)	NS	30 (20, 35)	30 (20, 40)	NS	10 (10, 10)	10 (0, 10)	NS
Vasoactive medications	15 (8.8%)	31 (9.0%)	NS	13 (50.0%)	26 (46.4%)	NS	2 (1.4%)	5 (1.7%)	NS
Intubation	14 (8.2%)	34 (9.8%)	NS	10 (38.5%)	20 (35.7%)	NS	4 (2.8%)	14 (4.8%)	NS
Outcome – All									
Paediatric Critical Care Unit admission	36 (21.1%)	80 (23.1%)	NS	18 (69.2%)	36 (64.3%)	NS	18 (12.4%)	44 (15.2%)	NS
Confirmed Bacteraemia (excluding contaminants)	22 (12.9%)	45 (13.0%)	NS	5 (19.2%)	15 (26.8%)	NS	17 (11.7%)	30 (10.3%)	NS
Sepsis recorded on discharge summary	73 (42.7%)	181 (52.3%)	*P* < 0.04	20 (76.9%)	41 (73.2%)	NS	53 (36.6%)	140 (48.3%)	*P* = 0.02
Length of stay *days*; median; (IQR)	4 (2, 8)	4 (3, 9)	NS	12 (6, 23)	8 (4, 22.5)	NS	4 (2, 7)	4 (3, 7)	NS
Readmitted within 30 days of discharge (planned and unplanned)	27 (15.8%)	78 (22.5%)	NS	5 (19.2%)	11 (19.6%)	NS	22 (15.2%)	67 (23.1%)	NS
30‐day disposition									
Discharged home	153 (89.5%)	315 (91.0%)	NS	19 (73.1%)	45 (80.4%)	NS	134 (92.4%)	270 (93.1%)	NS
Remained an inpatient	9 (5.3%)	9 (2.6%)		5 (19.2%)	2 (3.6%)		4 (2.8%)	7 (2.4%)	
Inpatient following readmission	7 (4.1%)	16 (4.6%)		1 (3.8%)	4 (7.1%)		6 (4.1%)	12 (4.1%)	
Deceased	2 (1.2%)	6 (1.7%)		1 (3.8%)	5 (8.9%)		1 (0.7%)	1 (0.3%)	

IQR, interquartile range.

Blood cultures were taken in 497 (96.1%) of the total cohort. Overall, 67 (13.0%) had confirmed bacteraemia, higher in the septic shock group (20/82; 24.4%) and those transferred for care (30/103; 29.1%). Common pathogens included *Streptococcus pyogenes* (*n* = 17), *Escherichia coli* (*n* = 11), *Staphylococcus aureus* (*n* = 9) and *Streptococcus pneumoniae* (*n* = 7).

The median length of hospital stay was 4 days (IQR: 3, 9) with no differences pre‐ and post‐pathway program implementation. For those with septic shock, median length of stay was 12 days pre‐ and 8 days post‐pathway, but this change was not statistically significant.

There were no significant differences detected between the pre‐ and post‐pathway periods for the 30‐day outcome. Eight children (1.5%) died within 30 days of admission (2/171; 1.2% pre‐pathway; 6/346; 1.7% post‐pathway). At 30 days, 90.5% of patients were discharged home, 3.5% remained hospitalised from their initial admission and 4.4% were in hospital following readmission.

There was an improvement in documentation of treatment for sepsis on discharge summaries (42.7% pre‐pathway; 52.3% post‐launch, *P* < 0.04).

### Pathway program impact on sepsis quality metrics (excluding transfers to PCH)

The pathway form was utilised for 53.9% (146/271) of episodes identified at PCH. Despite this, 94.1% (255/271) of episodes were managed according to the PCH Sepsis Recognition and Management Guideline regarding empiric antibiotic choice and timing. When not utilised, appropriate alternate clinical documentation forms were frequently used (e.g., resuscitation form; medical emergency response form).

The proportion of episodes with blood cultures collected, and collected prior to antibiotics, remained unchanged post‐pathway introduction (Table 3). However, the proportion of episodes where lactate was measured increased. The median lactate level across the total pre‐ and post‐pathway PCH cohort (*n* = 414) was 2.0 mmol/L (IQR: 1.3–3.0), while in the PCH septic shock cohort (*n* = 57), it was 3.1 mmol/L (IQR: 1.9–4.75) (Table 3). Forty‐two patients (10.1%) in the total PCH cohort had a lactate level exceeding 4.0 mmol/L.

**TABLE 3:** Quality Metrics (PCH escalations only) in the total cohort, those with septic shock and sepsis without shock, pre‐ and post‐pathway program

	Total cohort			Septic shock			Sepsis without shock		
	Pre‐pathway	Post‐pathway	Significance	Pre‐pathway	Post‐pathway	Significance	Pre‐pathway	Post‐pathway	Significance
	*n* = 143	*n* = 271		*n* = 20	*n* = 40		*n* = 123	*n* = 231	
Blood culture collection during admission	141 (98.6%)	264 (97.4%)	NS	20 (100%)	40 (100%)	NS	121 (98.4%)	224 (97%)	NS
Blood culture prior to antibiotics	116 (81.1%)	209 (77.1%)	NS	14 (70%)	28 (70%)	NS	102 (82.9%)	181 (78.4%)	NS
Lactate collection	122 (85.3%)	261 (96.3%)	*P* < 0.001	19 (95%)	38 (95%)	NS	103 (83.7%)	223 (96.5%)	*P* < 0.001
Result mmol/L; median; (IQR)	1.9 (1.3, 2.9)	2 (1.4, 3)	NS	2.2 (1.7, 5.4)	3.1 (2, 4.75)	NS	1.8 (1.2, 2.6)	1.9 (1.3, 2.8)	NS
Time to Ab minutes; median (IQR)	60 (26, 115)	45 (16, 75)	*P* < 0.001	38.5 (5.5, 71)	19.5 (7, 45)	NS	68 (30, 125)	48 (20, 82)	*P* < 0.01
Proportion receiving antibiotics within 60 min	73 (51.0%)	182 (67.2%)	*P* = 0.001	14 (70%)	37 (92.5%)	*P* < 0.03	59 (48.0%)	145 (62.8%)	*P* < 0.01
Proportion receiving antibiotics within 180 min	125 (87.4%)	259 (95.6%)	*P* = 0.002	19 (95%)	40 (100%)	NS	106 (86.2%)	219 (94.8%)	*P* = 0.005

**Figure 2:**
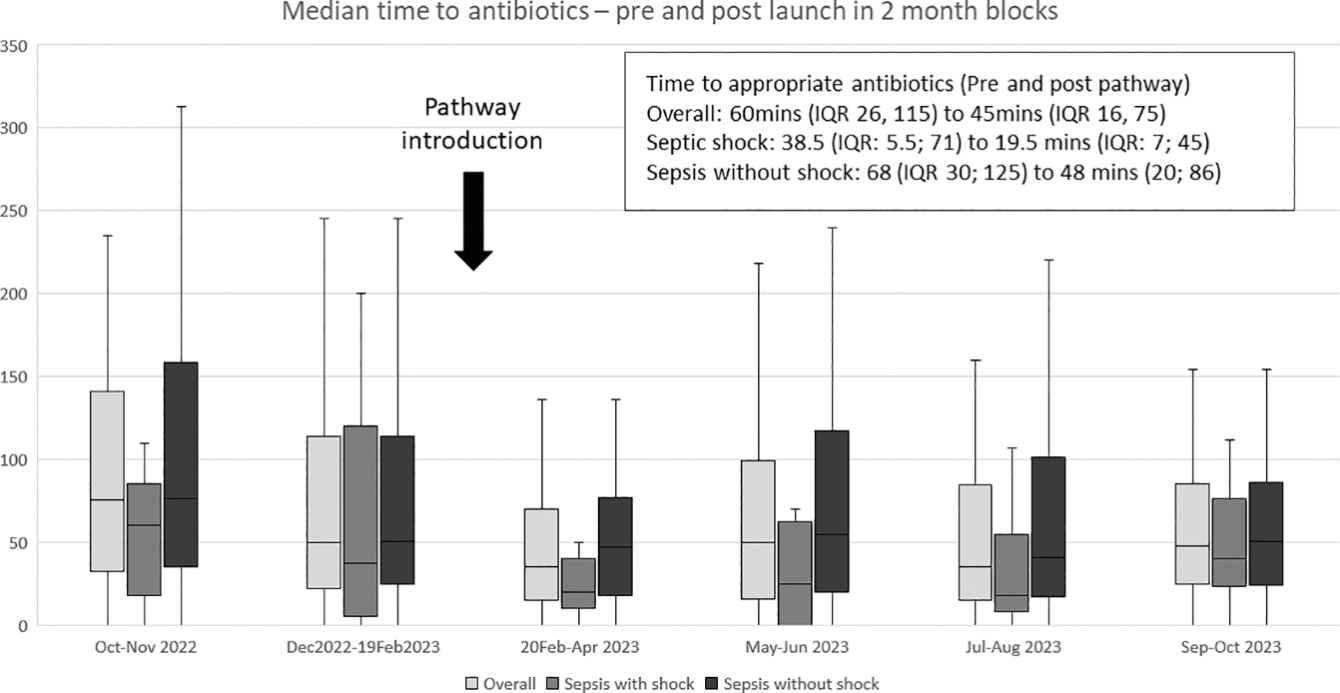
Box and whisker plot demonstrating a significant reduction in time (minutes) to appropriate antibiotic therapy following launch.

For the overall cohort, managed exclusively at PCH, the median time to appropriate empiric antibiotic therapy decreased from 60 (IQR: 26, 115) to 45 min (IQR: 16, 75; *P* < 0.001) post‐pathway (Table 3), a reduction sustained over the post‐pathway observation period (Fig. 2). In those with septic shock, the median time to antibiotics reduced from 38.5 (IQR: 5.5, 71) to 19.5 min (IQR: 7, 45; *P* = 0.24), while for sepsis without shock, it decreased from 68 (IQR: 30, 125) to 48 min (IQR: 20, 82; *P* < 0.001). The proportion of those receiving antibiotics for septic shock within 60 min increased from 70.0% to 92.5% (*P* < 0.03) and the proportion of those receiving antibiotics for sepsis without shock within 180 min increased from 86.2% to 94.8% (*P* = 0.005).

### Impact on antimicrobial use and workflow

A non‐statistically significant increase in antibiotic use was observed during the study period. Total hospital ceftriaxone use increased from 0.100 g/bed day (95% CI: 0.089; 0.110) pre‐launch to 0.127 g/bed day (95% CI: 0.102; 0.151) post‐launch. ED ceftriaxone use increased from 0.0158 (95% CI: 0.0129; 0.0179) to 0.0198 g/ED presentation (95% CI: 0.0164; 0.0224). No increase in antibiotic use was observed when the pre‐launch period (October 2022–January 2023) was compared with data obtained from October 2023 to January 2024.

## Discussion

Data from a tertiary paediatric hospital highlights the importance of a systematic approach to sepsis recognition, care, and monitoring. A locally developed pathway introduced with a corresponding education program led to an improvement in time to antibiotics and the proportion receiving antibiotics within recommended timeframes.

Paper pathway utilisation is impacted by several factors including knowledge, accessibility, and professional roles.^24^, ^25^ Utilisation must be prioritised through ongoing education to ensure familiarity with the pathway recognition and escalation processes and attempts to avoid duplication of clinical documentation. Because of the high number of sepsis mimics in children, it is important to balance the sensitivity and specificity of a sepsis pathway, ensuring it is tailored to local populations and workflows. ‘Over‐trigger’ of a pathway or excessive documentation can increase clinical workload, leading to poor clinician satisfaction and poor pathway utilisation. Monitoring over the first 5 weeks following pathway launch revealed the average number of ‘sepsis reviews’ in PCH ED was 2.1 per 100 overall presentations and 10.7 per 100 febrile presentations to the ED. Of those requiring sepsis reviews, an average of 14.6% received treatment for septic shock/sepsis without shock.

Our population had a median age of 1 year, consistent with previous studies.^4^ This median age and low bacteraemia rate (13.0%) highlight the challenge in distinguishing sepsis from self‐limiting febrile illnesses in acute settings, where a predominance of febrile viral presentations with abnormal physiology occurs in young children.^7^, ^26^ Bacteraemia rate rose to 24.4% for those treated for septic shock and 29.1% in those transferred for care. Paediatric sepsis is known to have a high culture‐negative rate, with a previous study showing 44.0% of patients admitted to intensive care with paediatric sepsis or septic shock having a bacterial pathogen identified.^27^ *Streptococcus pyogenes* was the most common pathogen, with increases in *S. pyogenes* cases noted both locally and globally.^28^

Aboriginal children were overrepresented, making up 13.0% of the total cohort despite comprising only 3.3% of the WA population.^29^ Aboriginal children have a higher risk of invasive infections, including bloodstream infections, pneumonia, and sepsis.^5^, ^30^ Risk factors for sepsis in these children relate to a complex interplay of factors including remoteness, access to culturally safe healthcare, socioeconomic resources, and historical and ongoing marginalisation.^5^

In regard to balancing measures, the incidence of sepsis with and without shock remained consistent pre‐and post‐pathway and there was no change in PCCU admission or overall mortality. There was no significant change in length of stay. Ceftriaxone use increased during the study period, but the change was not statistically significant and could not be clearly differentiated from seasonal variation in antibiotic use. It will be important to continue to monitor antimicrobial use as the program continues.

Sepsis pathway implementation provides nursing and medical staff with clear escalation pathways and can improve clinical communication and documentation in relation to paediatric sepsis. The paper pathway aids data collection to help provide a clearer picture of local epidemiology and outcomes and helps identify research opportunities, including in post‐sepsis care. Work is currently underway to roll out an adapted pathway to other sites across WA. Our experience supports the need for funded roles to successfully implement a pathway with an associated education program and data collection.

## Limitations

The present study was undertaken in a single institution over a single year. WA is the largest and most remote state in Australia. As such, our results may not be representative of other paediatric hospital settings. The present study identified a relatively small cohort, and some comparisons did not identify statistically significant differences. Given this and the lack of adjustment for multiple comparisons, both positive and negative results need to be interpreted cautiously. It is also possible that other unmeasured factors influenced improvements in quality metrics; however, there were no other identified events or changes in practice or policy to account for these differences.

While the definitions of sepsis, with and without shock, were pragmatic to capture patients treated hospital‐wide for sepsis/septic shock, we acknowledge they are not consistent with current published consensus definitions, which were unpublished at the time the present study was undertaken. Pragmatic definitions have been utilised in other paediatric sepsis research including recently conducted clinical trials.^19^, ^20^ Identification of ‘time point zero’ for clinical recognition was at times challenging because of inconsistent documentation and the retrospective nature of the study. If the ‘time point zero’ was identified from a physiological trigger or nursing escalation it would generally be an earlier time than a documented clinical review/impression by a doctor. We acknowledge that research extracting data from clinical records are subject to numerous potential biases. Although the present study did not employ double data extraction, we used a single clinical expert with additional experts used where additional clinical clarification was required or when the time points were unclear.^31^

The measures developed for this project to quantify antibiotic use, such as grams per presentation, are inherently complex because of the variability in patient size within the paediatric population. Additionally, the absence of a standardised system, such as an electronic medical record, for quantifying paediatric antibiotic use limits the comparability of this measure.

## Conclusion

The study provides evidence that sepsis pathways designed for local populations and workflows can aid sepsis management, specifically reducing time to antibiotic therapy and increasing the proportion of patients meeting time to antibiotic guidelines. A well‐designed paper pathway can incorporate data collection and surveillance to monitor performance against KPIs. Having clearly documented screening and ‘sepsis review’ times on the pathway improves the reliability of assessing key intervention timing. Performance improvements were seen regardless of utilisation of the paper pathway form, suggesting that the associated resourcing and education package to implement and sustain a pathway are crucial elements of any demonstrated improvements.

## Supporting information


**Data S1:** Supporting Information.

## Data Availability

The data that support the findings of the present study are available on request from the corresponding author. The data are not publicly available because of privacy or ethical restrictions.
